# Comparação entre Angiotomografia Coronariana e Angiografia Coronariana Invasiva na Doença Arterial Coronariana Não Obstrutiva: O Estudo *Brazilian Coronary ARtery Disease* (BARD)

**DOI:** 10.36660/abc.20250702

**Published:** 2026-04-01

**Authors:** Protásio Lemos Da Luz, Desiderio Favarato, Alexandre A. Abizaid, Luiz Antonio Machado César, Carlos Vicente Serrano, Antônio Carlos Palandri Chagas, Carlos E. Rochitte, Marco A. Gutierrez

**Affiliations:** 1 Hospital das Clínicas Faculdade de Medicina Universidade de São Paulo São Paulo SP Brasil Instituto do Coração do Hospital das Clínicas da Faculdade de Medicina da Universidade de São Paulo, São Paulo, SP – Brasil

**Keywords:** Aterosclerose, Isquemia, Diagnóstico, Infarto, Morte

## Abstract

**Fundamento:**

O prognóstico da doença arterial coronariana (DAC) não obstrutiva não está bem estabelecido. Dados comparativos entre angiotomografia coronariana (ATC) e angiografia coronariana invasiva (ACI) são limitados.

**Objetivos:**

Comparar as informações derivadas da ATC e da ACI na DAC não obstrutiva quanto à detecção e aos desfechos clínicos.

**Métodos:**

Acompanhamos 4.004 pacientes adultos que realizaram ACI (n = 2.355) ou ATC (n = 1.649) por uma mediana de 9 anos. O desfecho primário foi um composto de mortalidade por todas as causas, síndrome coronariana aguda/infarto agudo do miocárdio e acidente vascular cerebral. Foi realizado pareamento por escore de propensão para comparar os desfechos entre os grupos. O nível de significância foi fixado em 5%.

**Resultados:**

A taxa global de eventos foi de 6,9%. A ACI esteve associada a piores desfechos em comparação à ATC ( *hazard ratio* [HR] 0,54; intervalo de confiança [IC] 95% 0,42-0,68; p < 0,001). Pacientes com DAC não obstrutiva apresentaram piores desfechos do que aqueles sem DAC (HR 1,73; IC 95% 1,32-2,27; p < 0,001). Achados coronarianos normais na ATC estiveram associados a melhores desfechos do que achados normais na ACI (HR 0,39; IC 95% 0,24-0,62; p < 0,001). As taxas de eventos aumentaram proporcionalmente à carga de placa. Em 1.187 pares pareados, a ATC esteve associada à melhora da sobrevida (HR 0,57; IC 95% 0,42-0,78; p < 0,001).

**Conclusão:**

A ATC reflete mais de perto os desfechos clínicos do que a ACI. A DAC não obstrutiva apresenta risco substancial independentemente da modalidade de imagem.

## Graphical abstract

**Figura Central:**
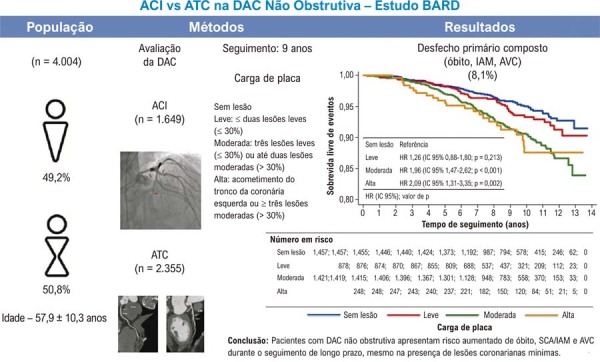
Comparação entre Angiotomografia Coronariana e Angiografia Coronariana Invasiva na Doença Arterial Coronariana Não Obstrutiva: O Estudo Brazilian Coronary ARtery Disease (BARD)

ACI: angiografia coronariana invasiva; ATC: angiotomografia coronariana; DAC: doença arterial coronariana; IAM: infarto agudo do miocárdio; AVC: acidente vascular cerebral; SCA: síndrome coronariana aguda; HR: hazard ratio; IC: intervalo de confiança.

## Introdução

Pacientes com doença arterial coronariana (DAC) obstrutiva (lesões ≥ 70%) têm sido amplamente estudados,^1^ enquanto pacientes com DAC não obstrutiva permanecem comparativamente menos investigados.^2 - 6^ No entanto, a DAC não obstrutiva é altamente prevalente e está associada a complicações clinicamente relevantes, incluindo mortalidade por todas as causas, infarto agudo do miocárdio (IAM) e acidente vascular cerebral (AVC), tanto em indivíduos sintomáticos quanto assintomáticos.^7 - 9^

A prevalência relatada de DAC não obstrutiva varia substancialmente. Em análises agrupadas, a prevalência global média foi de 67%, compreendendo 49% entre pacientes submetidos à ACI e 73% entre aqueles avaliados com angiotomografia coronariana (ATC). As taxas anualizadas de complicações foram de 0,7% na DAC não obstrutiva e 2,7% na DAC obstrutiva, o que indica que o risco global na DAC não obstrutiva é aproximadamente 72% menor do que na DAC obstrutiva.^6^ Essas complicações têm sido atribuídas principalmente à disfunção microvascular secundária à disfunção endotelial, ativação plaquetária e formação de trombo.^7 , 10 , 11^ No Brasil, dados comparativos de longo prazo entre ATC e ACI na DAC não obstrutiva permanecem limitados.

As modalidades de imagem mais comumente utilizadas para o diagnóstico de DAC são a angiografia coronariana invasiva (ACI) e a ATC. Embora essas modalidades diferenciem-se substancialmente, poucos estudos as compararam diretamente. O estudo DISCHARGE^12^ aleatorizou 3.561 pacientes para ACI ou ATC e não relatou diferenças significativas nas taxas de eventos entre ATC (2,1%) e ACI (3,0%) ao longo de um seguimento mediano de 3,5 anos. Por outro lado, uma metanálise de Xie et al.,^13^ incluindo mais de 26.000 pacientes com DAC estável em seis estudos, sugeriu que a ACI está associada a maiores taxas de eventos cardiovasculares adversos maiores (ECAMs) e mortalidade por todas as causas em comparação com a ATC.

À luz desses achados conflitantes, este estudo foi delineado para avaliar o desempenho comparativo da ACI e da ATC em uma grande coorte de pacientes com DAC não obstrutiva durante seguimento de longo prazo, com ênfase nos desfechos clínicos e nos fatores associados ao prognóstico.

## Métodos

### População

Entre 2011 e 2017, foram avaliados 4.004 pacientes consecutivos com idade entre 18-80 anos, homens e mulheres, provenientes de uma instituição especializada em cardiologia. Todos os participantes não apresentavam eventos coronarianos prévios e foram encaminhados por sintomas anginosos (41%), testes funcionais positivos para isquemia (17,5%), *check-up* de rotina (23%) ou avaliação pré-operatória (1,6%); em 16,9% dos casos, a indicação não estava disponível. Os pacientes foram provenientes do registro hemodinâmico do Instituto do Coração e selecionados por conveniência.

Os critérios de exclusão incluíram síndrome coronariana aguda (SCA)/IAM prévios, revascularização miocárdica prévia, doença valvar, cardiomiopatias, doença pulmonar obstrutiva crônica, doença renal crônica, câncer e insuficiência hepática.

O desfecho primário foi um composto de mortalidade por todas as causas, SCA/IAM e AVC. Os desfechos secundários compreenderam os componentes individuais do desfecho primário. Para reduzir confundimento nas comparações entre ACI e ATC, foi realizado pareamento por escore de propensão.^14^

O estudo foi aprovado pelo Comitê de Ética em Pesquisa com Seres Humanos institucional do Hospital das Clínicas da Faculdade de Medicina da Universidade de São Paulo e registrado no ClinicalTrials.gov (NCT05392491).

A ACI e a ATC foram realizadas por operadores treinados utilizando técnicas padronizadas. Cada caso foi revisado de forma independente por dois técnicos treinados que analisaram relatórios gerados por dois angiografistas experientes responsáveis pela interpretação das imagens. Em casos de discordância, os registros foram revisados por dois cardiologistas seniores para obtenção de consenso. A variabilidade interobservador foi de 2,2% em 500 casos selecionados aleatoriamente utilizando sequência gerada por computador (random.org). Não ocorreram complicações relacionadas aos procedimentos.

O seguimento foi realizado por meio de consultas ambulatoriais, contato telefônico ou e-mail. Após consentimento informado, os participantes responderam a um questionário estruturado ( Apêndice A ). A adjudicação dos desfechos foi baseada em relatos dos pacientes, relatos familiares, prontuários médicos ou documentação do médico assistente.

As artérias coronárias foram classificadas como:

- Ausência aparente de DAC;
- Presença de DAC, classificada de acordo com uma abordagem CAD-RADS™ 2.0 modificada adaptada para DAC não obstrutiva. A gravidade das lesões foi definida como: ausente (0%), leve (≤ 30%) e moderada (> 30%).

O índice de carga de placa foi categorizado como:

- Ausente: nenhuma lesão;
- Leve: até duas lesões leves (≤ 30%);
- Moderada: três lesões leves (≤ 30%) ou até duas lesões moderadas (> 30%);
- Alta: acometimento do tronco da coronária esquerda ou pelo menos três lesões moderadas (> 30%);
- Muito alta: carga de lesão maior do que a previamente classificada como alta, ou pelo menos uma lesão ≥ 50% identificada em ATC subsequente quando disponível.

As lesões coronarianas foram definidas visualmente como o diâmetro luminal do vaso doente comparado ao segmento proximal de aparência mais normal. Não ocorreram complicações durante a ATC.

### Dados demográficos

Os dados basais coletados na inclusão do estudo ( Tabela 1 ) incluíram idade, sexo, peso, altura e pressão arterial. O índice de massa corporal (IMC) foi calculado como peso dividido pela altura ao quadrado.

**Tabela 1:** – Características basais de acordo com a modalidade de imagem (ACI vs ATC)

Variável	ACI (n = 1.649)	ATC (n = 2.355)	Valor p	Total (%)
**Sexo, n (%)**				
Mulheres	956 (58,0)	1.080 (45,9)	< 0,001	2.036 (50,8)
Homens	693 (42,0)	1.275 (54,1)	< 0,001	1.968 (49,2)
**Raça, n (%)**				
Branca	1.148 (86,5)	1.790 (89,9)	> 0,05	3.208 (87,8)
**Idade, n (%)**				
≤ 65 anos	1.263 (76,6)	1.857 (78,9)	0,008	3.120 (77,9)
> 65 anos	386 (23,4)	497 (21,1)	0,008	883 (22,1)
**Fatores de risco cardiovascular, n (%)**				
História familiar de DAC	703 (52,9)	1.139 (57,0)	0,022	1.842 (55,4)
Diabetes melito	689 (41,8)	847 (36,0)	< 0,001	1.536 (38,4)
Dislipidemia	1.082 (65,6)	1.690 (71,8)	< 0,001	2.772 (69,2)
HAS	1.355 (82,2)	1.673 (71,0)	< 0,001	3.028 (75,6)
Tabagismo	710 (44,7)	936 (41,1)	0,027	1.646 (42,5)
Consumo de álcool	358 (33,8)	910 (52,9)	0,001	1.268 (45,6)
Exercício físico	650 (41,7)	1.188 (53,1)	< 0,001	1.838 (48,4)
Obesidade	434 (29,4)	594 (26,8)	0,001	1.028 (27,8)

ACI: angiografia coronariana invasiva; ATC: angiotomografia coronariana; DAC: doença arterial coronariana; HAS: hipertensão arterial sistêmica.

Hipertensão foi definida como pressão arterial sistólica > 130/85 mmHg ou uso de medicação anti-hipertensiva. Obesidade foi definida como IMC ≥ 30, e sobrepeso como IMC de 26-29.

A atividade física foi categorizada como sedentário ou ativo (≥ três sessões por semana). História familiar positiva foi definida como pelo menos um dos pais com eventos cardiovasculares ou intervenções antes dos 60 anos.

O status tabágico foi classificado como nunca, ex-fumante ou atual (≥ 10 cigarros/dia). Diabetes mellitus foi definido como glicemia de jejum ≥ 126 mg/dL, teste de tolerância à glicose de 2 horas > 200 mg/dL, HbA1c ≥ 6,5% ou uso de medicação hipoglicemiante. Dislipidemia foi definida como LDL > 130 mg/dL, triglicerídeos > 150 mg/dL ou uso de terapia hipolipemiante.

Os dados laboratoriais são apresentados na Tabela 2 . Não foram observadas diferenças significativas entre os grupos para nenhum parâmetro, exceto colesterol total.

**Tabela 2:** – Dados laboratoriais de acordo com a modalidade de imagem (ACI vs ATC)

Variável (média ± DP)	ACI (n = 1.649)	ATC (n = 2.355)	Valor p	Total
**Ureia (mg/dl)**	36,33 ± 11,46 (n = 732)	35,52 ± 10,24 (n = 1.187)	0,435	35,83 ± 10,72 (n = 1.919)
**Creatinina (mg/dl)**	0,95 ± 0,26 (n = 808)	0,94 ± 0,21 (n = 1.231)	0,922	0,94 ± 0,23 (n = 2.039)
**Sódio (mEq/l)**	140,36 ± 2,49 (n = 639)	140,30 ± 2,42 (n = 1.086)	0,687	140,32 ± 2,44 (n = 1.725)
**Potássio (mEq/l)**	4,32 ± 0,44 (n = 642)	4,32 ± 0,40 (n = 1.088)	0,612	4,32 ± 0,41 (n = 1.730)
**Colesterol total (mg/dl)**	190,09 ± 42,30 (n = 740)	196,93 ± 49,63 (n = 1.114)	0,043	194,20 ± 46,95 (n = 1.854)
**HDL-C (mg/dl)**	47,94 ± 14,19 (n = 738)	49,07 ± 14,29 (n = 1.113)	0,055	48,62 ± 14,26 (n = 1.851)
**Colesterol não-HDL (mg/dl)**	142,30 ± 41,33 (n = 737)	147,84 ± 49,21 (n = 1.113)	0,153	145,63 ± 46,30 (n = 1.850)
**LDL-C (mg/dl)**	115,39 ± 37,97 (n = 724)	119,88 ± 44,80 (n = 1.085)	0,273	118,08 ± 42,25 (n = 1.809)
**Triglicerídeos (mg/dl)**	133,64 ± 75,73 (n = 721)	143,75 ± 170,51 (n = 1.129)	0,489	139,81 ± 141,40 (n = 1.850)
**Glicose (mg/dl)**	113,77 ± 34,18 (n = 751)	113,11 ± 37,79 (n = 1.122)	0,045	113,37 ± 36,38 (n = 1.873)
**HbA1c (%)**	6,30 ± 1,28 (n = 481)	6,00 ± 1,04 (n = 919)	< 0,001	6,10 ± 1,14 (n = 1.400)

ACI: angiografia coronariana invasiva; ATC: angiotomografia coronariana; DP: desvio padrão; HDL-C: colesterol de lipoproteína de alta densidade; LDL-C: colesterol de lipoproteína de baixa densidade.

### Análise estatística

Variáveis categóricas são apresentadas como frequências e comparadas pelo teste do qui-quadrado. Variáveis contínuas com distribuição normal são expressas como média ± desvio padrão e comparadas por testes *t* não pareados. A normalidade foi avaliada por testes de Kolmogorov-Smirnov e inspeção de histogramas.

O tempo desde o exame basal até o desfecho primário foi calculado para análises de sobrevida. A sobrevida livre de eventos foi estimada por curvas de Kaplan-Meier. Análises de regressão de Cox univariada e multivariável foram realizadas quando apropriado, e os resultados são apresentados como *hazard ratios* (HRs) com intervalos de confiança (ICs) de 95%.

Para controle adicional de confundidores, foi aplicado escore de propensão baseado em regressão logística utilizando caliper de 0,01.^14^ O pareamento incluiu nove covariáveis: idade, sexo, pressão arterial, dislipidemia, tabagismo, atividade física, diabetes, hipertensão e história familiar de DAC.

Um nível de significância de 5% foi adotado para todas as análises. As análises estatísticas foram realizadas utilizando o IBM SPSS Statistics for Windows, versão 25 (IBM Corp., Armonk, N.Y., EUA).

## Resultados

### Características basais

As características demográficas basais estão resumidas na Tabela 1 . Um total de 4.004 pacientes foi acompanhado por uma mediana de 9 anos. A idade média foi 57,9 ± 10,3 anos. No geral, homens e mulheres estiveram representados de forma semelhante; no entanto, uma maior proporção de homens foi observada no grupo ATC (54,1% vs 42,0%). A ATC foi realizada em 2.355 pacientes (58,8%) e a ACI em 1.649 (41,2%).

História familiar positiva de DAC, dislipidemia e atividade física foram mais frequentes no grupo ATC, enquanto diabetes, hipertensão e tabagismo foram mais prevalentes no grupo ACI. Carga de placa moderada ou maior na linha de base foi mais comum entre pacientes submetidos à ACI do que entre pacientes submetidos à ATC (68,2% vs 23,1%; p < 0,001, teste do qui-quadrado) ( Apêndice B ).

### Número total de eventos

Durante o seguimento, ocorreram 278 eventos (6,9%) ao longo de 9 anos, com maior incidência no grupo ACI em comparação ao grupo ATC (9,4% vs 5,1%), predominantemente entre homens com mais de 65 anos. A diferença absoluta nas taxas de eventos foi de 4,3%.

Óbito, AVC e SCA/IAM foram os eventos mais frequentes na população geral e foram significativamente mais comuns no grupo ACI do que no grupo ATC ( Tabela 3 ).

**Tabela 3:** – Número de eventos clínicos de acordo com a modalidade de imagem (ACI vs ATC)

Desfecho	ACI n (%) (n = 1.649)	ATC n (%) (n = 2.355)	Total n (%) (n = 4.004)	Valor p
**Desfecho primário composto**	155 (9,4)	121 (5,1)	276 (6,9)	< 0,001
**Desfechos isolados**				
Óbito	72 (4,4)	44 (1,9)	116 (2,9)	< 0,001
AVC	65 (3,9)	47 (2,0)	111 (2,8)	< 0,001
SCA/IAM	49 (3,0)	39 (1,7)	88 (2,2)	0,005

Componentes individuais do desfecho primário. ACI: angiografia coronariana invasiva; ATC: angiotomografia coronariana; AVC: acidente vascular cerebral; SCA: síndrome coronariana aguda; IAM: infarto agudo do miocárdio.

### Sobrevida e risco de eventos em pacientes com e sem doença arterial coronariana

**Figura 1:**
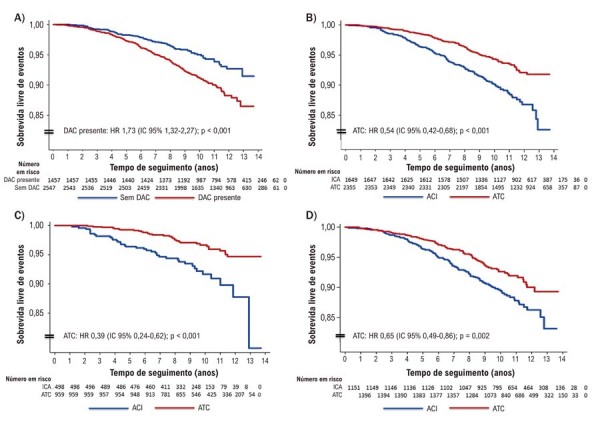
– Sobrevida livre de eventos de acordo com o status de DAC e a modalidade de imagem (ACI vs ATC). A) Sobrevida livre de eventos de acordo com o status de DAC; B) sobrevida livre de eventos de acordo com a modalidade de imagem; C) sobrevida livre de eventos entre pacientes sem DAC aparente; D) sobrevida livre de eventos entre pacientes com DAC não obstrutiva. ACI: angiografia coronariana invasiva; ATC: angiotomografia coronariana; DAC: doença arterial coronariana; HR: hazard ratio; IC: intervalo de confiança.

No geral, 37,3% dos pacientes não apresentavam DAC aparente e 62,7% apresentavam DAC não obstrutiva. A DAC não obstrutiva esteve associada a sobrevida significativamente pior em comparação à ausência de DAC ( Figura 1A ). A sobrevida livre de eventos também foi pior entre pacientes ACI do que entre pacientes ATC ( Figura 1B ).

Notavelmente, pacientes ACI sem DAC aparente apresentaram sobrevida significativamente pior do que pacientes comparáveis no grupo ATC ( Figura 1C ), sugerindo que as duas modalidades fornecem informações prognósticas diferentes, apesar da classificação semelhante como normal ou ausência aparente de DAC.

Além disso, entre pacientes com DAC não obstrutiva identificada por ACI ou ATC, a sobrevida permaneceu melhor no grupo ATC em comparação ao grupo ACI ( Figura 1D ).

### Índice de carga de placa vs eventos clínicos

**Figura 2:**
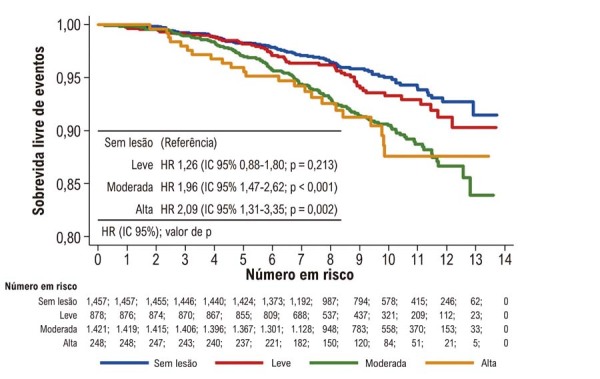
– Sobrevida livre de eventos de acordo com o índice de carga de placa. HR: hazard ratio; IC: intervalo de confiança.

A Figura 2 ilustra a relação entre carga de placa e eventos clínicos. Não foram observadas diferenças estatisticamente significativas entre pacientes com baixa carga de placa e aqueles sem lesões detectáveis ao longo do seguimento.

Em contraste, pacientes com carga de placa moderada e alta apresentaram risco significativamente maior para o desfecho primário em comparação àqueles sem lesões, com risco semelhante entre os grupos de carga moderada e alta.

### Medicações

A terapia hipolipemiante foi utilizada com maior frequência no grupo ATC, enquanto agentes antidiabéticos, medicamentos anti-hipertensivos e aspirina foram mais comumente utilizados no grupo ACI. Resultados detalhados são apresentados no Apêndice C .

### Determinantes da evolução clínica

Análises não ajustadas identificaram 16 variáveis associadas aos desfechos. Após ajuste, sete variáveis permaneceram independentemente associadas aos eventos (p < 0,001): sexo masculino, hipertensão, idade > 65 anos, alocação no grupo ACI, tabagismo, inatividade física, história familiar positiva de DAC e carga de placa.

### Pareamento por escore de propensão

Como este não foi um estudo randomizado, foi realizado escore de propensão utilizando idade, sexo, pressão arterial sistólica, dislipidemia, tabagismo, atividade física, diabetes, hipertensão e história familiar de DAC ( Tabela 4 ). Um total de 1.186 pares pareados foi identificado.

**Tabela 4:** – Características basais após pareamento por escore de propensão (ACI vs ATC)

Característica	AC (n = 1.187), n (%)	ATC (n = 1.187), n (%)	Valor p^**1**^
**Sexo, n (%)**			0,019
Feminino	663 (55,9)	637 (53,7)	
Masculino	527 (44,1)	550 (46,3)	
**Idade, n (%)**			0,955
≤ 65 anos	920 (77,5)	918 (77,3)	
> 65 anos	267 (22,5)	269 (22,7)	
**Fatores de risco cardiovascular, n (%)**			
História familiar de DAC	631 (53,2)	636 (53,6)	0,828
Diabetes melito	465 (39,2)	499 (42,0)	0,080
Dislipidemia	796 (67,1)	808 (68,1)	0,524
HAS	959 (80,8)	967 (81,5)	0,546
Tabagismo	511 (43,0)	484 (40,8)	0,155
Exercício físico	504 (42,5)	469 (39,5)	0,055

^1^Teste de McNemar para amostras pareadas. ACI: angiografia coronariana invasiva; ATC: angiotomografia coronariana; DAC: doença arterial coronariana; HAS: hipertensão arterial sistêmica.

Entre os pacientes pareados, o sexo masculino permaneceu mais prevalente no grupo ACI, enquanto história familiar positiva foi mais frequente no grupo ATC. Todas as demais variáveis ficaram equilibradas entre os grupos.

Após o pareamento, a sobrevida permaneceu menor no grupo ACI em comparação ao grupo ATC (HR 0,57; IC 95% 0,42-0,78; p < 0,001), sugerindo que pacientes submetidos à ACI apresentavam DAC mais avançada.

## Discussão

Neste estudo observacional de longo prazo, ACI e ATC forneceram informações prognósticas diferentes na DAC não obstrutiva, que esteve associada a eventos clinicamente relevantes. Como esperado, pacientes sem DAC apresentaram melhores desfechos do que aqueles com DAC não obstrutiva, independentemente da modalidade de imagem. Esses achados corroboram estudos prévios que demonstram que a DAC não obstrutiva está associada a complicações substanciais, incluindo eventos cardiovasculares, hospitalizações recorrentes e pior qualidade de vida.^15 - 17^

Notavelmente, pacientes sem DAC aparente no grupo ACI apresentaram pior prognóstico do que pacientes comparáveis no grupo ATC. Além disso, em toda a coorte, indivíduos submetidos à ACI demonstraram piores desfechos do que aqueles avaliados com ATC. Esses resultados são consistentes com evidências prévias. Huang et al.^16^ analisaram 64.905 pacientes submetidos à ACI ou ATC, e Radico et al.^18^ avaliaram 34.039 pacientes com angina e DAC não obstrutiva ao longo de 5 anos; ambos os estudos concluíram que a DAC não obstrutiva está associada a maiores taxas de MACE em comparação com artérias coronárias completamente normais. De fato, Alfonso et al.^19^ demonstraram, por meio de ultrassonografia intravascular, que vasos considerados normais pela ACI podem apresentar placas ateroscleróticas. Além disso, Ludmer et al.^20^ mostraram que pacientes com doença coronariana mínima exibem vasoconstrição paradoxal em resposta à acetilcolina, indicando disfunção endotelial como provável mecanismo subjacente aos eventos em pacientes com coronárias angiograficamente normais.^21 , 22^

Nossos achados também são consistentes com a metanálise de Xie et al.,^13^ que incluiu 26.548 pacientes de seis estudos comparando ACI e ATC. Os autores relataram que a ACI esteve associada a maiores riscos de MACE, mortalidade por todas as causas e complicações maiores relacionadas ao procedimento em comparação com a ATC, particularmente durante seguimento mais curto (< 3 anos). No entanto, nossos resultados diferem dos do estudo DISCHARGE,^12^ que não encontrou diferença nas taxas de eventos entre ACI e ATC ao longo de um seguimento mediano de 3,5 anos. Embora nosso estudo não tenha sido randomizado, o seguimento substancialmente mais longo (9 vs 3,5 anos) pode explicar essa discrepância. Importante destacar que a análise por escore de propensão sugere que as diferenças entre as modalidades não foram totalmente explicadas pelos confundidores mensurados, exceto história familiar positiva, mais comum no grupo ATC, e sexo masculino, mais prevalente no grupo ACI. Ainda assim, confundimento residual não pode ser excluído, especialmente porque a escolha da modalidade de imagem foi determinada por julgamento clínico, provavelmente favorecendo ACI em pacientes com doença mais avançada considerados para revascularização.

A discrepância entre ACI e ATC observada neste estudo provavelmente reflete diferenças intrínsecas entre as modalidades. A ACI identifica principalmente estreitamento luminal, enquanto a ATC detecta alterações ateroscleróticas mais precoces, incluindo remodelamento positivo e calcificação coronariana, além da estenose luminal. Não foi realizada imagem intracoronária ou avaliação fisiológica. Embora tais informações fossem valiosas, essas técnicas não são rotineiramente utilizadas na prática clínica diária.

O índice de carga de placa esteve fortemente associado aos eventos clínicos. Pacientes sem DAC aparente ou com baixa carga de placa apresentaram desfechos semelhantes, enquanto carga de placa moderada e alta estiveram claramente associadas a pior prognóstico ( Figura 2 ). Esses achados estão alinhados a estudos prévios que demonstram que o risco de eventos aumenta proporcionalmente ao número de vasos acometidos, incluindo territórios extracoronarianos, como as artérias carótidas.^23 - 25^ Em nossa coorte, maior carga de placa esteve associada a idade mais avançada (>65 anos), consistente com observações prévias na DAC obstrutiva.^5^ Em contraste com alguns relatos anteriores, a DAC não obstrutiva foi mais frequente em homens do que em mulheres em nossa coorte, o que pode refletir características específicas da população.

Quanto à terapia farmacológica, medicamentos hipolipemiantes foram utilizados com maior frequência no grupo ATC, enquanto agentes antidiabéticos, fármacos anti-hipertensivos e aspirina foram mais comuns no grupo ACI. O uso de medicamentos pode ter influenciado os desfechos; no entanto, dada a natureza autorreferida desses dados e o longo período de seguimento, efeitos específicos não podem ser determinados. Fatores de risco tradicionais, incluindo dislipidemia, hipertensão, tabagismo e inatividade física, estiveram presentes em mais da metade da população, reforçando seu papel como determinantes da aterosclerose subclínica. Assim como os dados de medicação, essas variáveis devem ser interpretadas com cautela devido à dependência de autorrelato de longo prazo.

### Pontos fortes e limitações

Os principais pontos fortes deste estudo incluem o grande tamanho amostral e o longo seguimento observacional (mediana de 9 anos), que, até onde sabemos, representa um dos períodos de acompanhamento mais longos relatados nesse contexto. O desfecho primário incluiu apenas desfechos duros: mortalidade por todas as causas, AVC e SCA/IAM. Além disso, este estudo pragmático reflete a prática clínica do mundo real ao avaliar ACI e ATC, modalidades de imagem rotineiramente utilizadas na cardiologia diária, aumentando a relevância clínica dos achados.

Diversas limitações devem ser reconhecidas. Os dados foram autorreferidos, introduzindo potencial viés, e o desenho de centro único pode limitar a generalização. Embora escore de propensão tenham sido utilizados para mitigar confundimento, a ausência de randomização permanece uma limitação importante. Em particular, a escolha da modalidade de imagem pelo médico assistente pode ter influenciado os desfechos. Além disso, a ausência de exames de imagem repetidos impediu a avaliação da progressão da aterosclerose ou da instabilidade de placa, o que pode ter contribuído para os eventos clínicos.

## Conclusão

De modo geral, nossos achados destacam diferenças importantes entre ACI e ATC na DAC não obstrutiva durante o seguimento de longo prazo e confirmam que a DAC não obstrutiva está associada a risco clínico significativo. Pacientes com DAC não obstrutiva não devem ser tranquilizados como se fossem normais ou apresentassem aterosclerose clinicamente insignificante. A escolha da modalidade de imagem deve ser individualizada de acordo com o julgamento clínico. Testes funcionais de isquemia e estratégias preventivas agressivas devem ser priorizados nessa população. O controle precoce dos fatores de risco, por meio de terapia farmacológica e modificação do estilo de vida, é essencial.

## Material suplementar

Apêndice A

Apêndice B

Apêndice C
