# Supplementary material for: Comparação entre Angiotomografia Coronariana e Angiografia Coronariana Invasiva na Doença Arterial Coronariana Não Obstrutiva: O Estudo Brazilian Coronary ARtery Disease (BARD)
Source: Arq Bras Cardiol. 2026 Apr 1;123(3):e20250702. [Article in Portuguese] doi: 10.36660/abc.20250702 (PMC13128201; doi:10.36660/abc.20250702)
Supplement: Apêndice A [file 0066-782x-abc-123-3-e20250702-suppl01.pdf]

APPENDIX A - BARD QUESTIONAIRE

DEMOGRAPHICS

- Name
- Hospital registry - number
- Entry into study - date
- Birthday
- Age and sex
- Identification number
- Ethnics (White, black and other)
- Health provider
- Mother's name and ID number
- Address

CONTACT

- Telephone complete (state code, number)
- Prefered contac form (e-mail, telephone and whatsapp)
- Informed consente by telphone
- Person who answered questonaire

INITIAL ICA/CTA

- Exam performed: ICA (invasive angiography); TCA (computed angiotomograpy)
- Reason for the test:
    a) Suspected CAD
    b) Anginal pain
- Date of examen

CLINICAL DATA

- Family history for CAD
- Arrhythmias
- Diabetes
- Dyslipidemia
- Arterial Hypertension

- Heart Failure
- Renal disease
- Valve diaseses
- Mitral prolapse
- Pacemaker / ICD
- Echocardiogram
- Cerebral vascular accident
- Weight and height

- Physical Exercise? (yes or no, frequency/week; type of exercise(aerobic, strength)
- Alcohol consumation
  - Yes/No
  - Frequency/week
  - Type (wine, beer, spirits)
  - Ex-drinker
- Smoking
  - Yes/No
  - Cigarretes/day; if yes, how long
  - Ex-smoker

ICA/CTA - Evolution

- Was a new examen performed?
- Which examen:
  - ICA/CTA
- Reason for new examen
- Date of examen

CLINICAL EVOLUTION

- Last visit at InCor
- Last contact by phone and e-mail
- Follow up (attending physician, other)
- Present status

- Survival (Years)
- Death (date, cause)
- Cardiovascular events: AMI/ACS; coronary angioplasty; pacemaker; ICD; hospitalization; cardiac revascularization (CABG or PCI)

Obs: Data obtained by questionaire were tabulated for each patient for subsequent analysis in the Redcap program. Data are enclosed in appendix B. All data can be assessed through Redcap.
