# Supplementary material for: Comparação entre Angiotomografia Coronariana e Angiografia Coronariana Invasiva na Doença Arterial Coronariana Não Obstrutiva: O Estudo Brazilian Coronary ARtery Disease (BARD)
Source: Arq Bras Cardiol. 2026 Apr 1;123(3):e20250702. [Article in Portuguese] doi: 10.36660/abc.20250702 (PMC13128201; doi:10.36660/abc.20250702)
Supplement: Apêndice B [file 0066-782x-abc-123-3-e20250702-suppl02.pdf]

APPENDIX B – PLAQUE BURDEN INDEX BASED ON CAD-RADS 2.0 IN ICA AND CTA

	ICA (n=1649) n(%)	CTA (n=2355) n(%)	Total (n = 4004) n(%)	p
**Plaque burden**				<0.001[1]
Non- apparent/no CAD	498 (30.2%)	959 (40.7%)	1457 (36.4%)	
Mild	27 (1.6%)	851 (36.1%)	878 (21.9%)	
Moderate	1088 (66.0%)	333 (14.1%)	1421 (35.5%)	
Higher	36 (2.2%)	212 (9.0%)	248 (6.2%)	

[1] Pearson's chi square test.
ICA = Invasive coronary angiography; CTA = Coronary computed tomography
