# Supplementary material for: Comparação entre Angiotomografia Coronariana e Angiografia Coronariana Invasiva na Doença Arterial Coronariana Não Obstrutiva: O Estudo Brazilian Coronary ARtery Disease (BARD)
Source: Arq Bras Cardiol. 2026 Apr 1;123(3):e20250702. [Article in Portuguese] doi: 10.36660/abc.20250702 (PMC13128201; doi:10.36660/abc.20250702)
Supplement: Apêndice C [file 0066-782x-abc-123-3-e20250702-suppl03.pdf]

APPENDIX C – MEDICATIONS USED DURING FOLLOW-UP

		ICA (n=1649)	CTA (n=2355)	Total (n=4004)	P-value[1]
**Anti-lipidemics (High intensity statin, fibrates)**	Yes	919 (63.5%)	1536 (73.9%)	2455 (69.6%)	<0.001
	No	529 (36.5%)	543 (26.1%)	1072 (30.4%)	
	Missing	201	276	477	
**Anti-diabetics (Insulin, GLP1 agonists, SGLT2 antagonists)**	Yes	619 (42.8%)	794 (38.2%)	1413 (40.1%)	0.006
	No	828 (57.2%)	1284 (61.8%)	2112 (59.9%)	
	Missing	202	277	479	
**Anti-hypertensives (ACE inibitors, ARBs)**	Yes	1278 (77.5%)	1610 (68.4%)	2888 (72.1%)	<0.001
	No	371 (22.5%)	745 (31.6%)	1116 (27.9%)	
**Aspirin**	Yes	468 (32.3%)	575 (27.7%)	2482 (70.4%)	0.003
	No	980 (67.7%)	1502 (72.3%)	1043 (29.6%)	
	Missing	201	278	47	

Legend: Comparison of medications between ICA and CTA groups.
[1] Pearson's chi square test.
