# Supplementary material for: Comparação entre Angiotomografia Coronariana e Angiografia Coronariana Invasiva na Doença Arterial Coronariana Não Obstrutiva: O Estudo Brazilian Coronary ARtery Disease (BARD)
Source: Arq Bras Cardiol. 2026 Apr 1;123(3):e20250702. [Article in Portuguese] doi: 10.36660/abc.20250702 (PMC13128201; doi:10.36660/abc.20250702)
Supplement: Translation

# Translation: Comparison of Coronary Computed Tomography Angiography and Invasive Coronary Angiography in Nonobstructive Coronary Artery Disease: The Brazilian Coronary ARtery Disease (BARD) Study

**DOI:** 10.36660/abc.20250702i

**Authors:** Protásio Lemos Da Luz, Desiderio Favarato, Alexandre A. Abizaid, Luiz Antonio Machado César, Carlos Vicente Serrano, Antônio Carlos Palandri Chagas, Carlos E. Rochitte, Marco A. Gutierrez

**Affiliations:** 1 Brazil Instituto do Coração do Hospital das Clínicas da Faculdade de Medicina da Universidade de São Paulo, São Paulo, SP – Brazil

**Keywords:** Atherosclerosis, Ischemia, Diagosis, Infarction, Death

## Abstract

**Background:**

The prognosis of nonobstructive coronary artery disease (CAD) is not well established. Comparative data between coronary computed tomography angiography (CCTA) and invasive coronary angiography (ICA) are limited.

**Objectives:**

To compare CCTA- and ICA-derived information in nonobstructive CAD regarding detection and clinical outcomes.

**Methods:**

We followed 4,004 adult patients who underwent either ICA (n = 2,355) or CCTA (n = 1,649) for a median of 9 years. The primary endpoint was a composite of all-cause mortality, acute coronary syndrome/acute myocardial infarction, and stroke. Propensity score matching was performed to compare outcomes between groups. The significance level was set at 5%.

**Results:**

The overall event rate was 6.9%. ICA was associated with worse outcomes than CCTA (hazard ratio [HR] 0.54; 95% CI 0.42-0.68; p < 0.001). Patients with nonobstructive CAD had worse outcomes than those without CAD (HR 1.73; 95% CI 1.32-2.27; p < 0.001). Normal coronary findings on CCTA were associated with better outcomes than normal findings on ICA (HR 0.39; 95% CI 0.24-0.62; p < 0.001). Event rates increased proportionally with plaque burden. In 1,187 matched pairs, CCTA was associated with improved survival (HR 0.57; 95% CI 0.42-0.78; p < 0.001).

**Conclusion:**

CCTA more closely reflects clinical outcomes than ICA. Nonobstructive CAD carries a substantial risk regardless of imaging modality.

## Introduction

Patients with obstructive coronary artery disease (CAD; lesions ≥ 70%) have been extensively studied,^1^whereas patients with nonobstructive CAD remain comparatively underinvestigated.^2 - 6^Nevertheless, nonobstructive CAD is highly prevalent and associated with clinically relevant complications, including all-cause mortality, acute myocardial infarction (AMI), and stroke, in both symptomatic and asymptomatic individuals.^7 - 9^

The reported prevalence of nonobstructive CAD varies substantially. In pooled analyses, the overall prevalence averaged 67%, comprising 49% among patients undergoing ICA and 73% among those evaluated with coronary computed tomography angiography (CCTA). Annualized complication rates were 0.7% in nonobstructive CAD and 2.7% in obstructive CAD, which indicates that overall risk in nonobstructive CAD is approximately 72% lower than in obstructive CAD.^6^These complications have been primarily attributed to microvascular dysfunction secondary to endothelial dysfunction, platelet activation, and thrombus formation.^7 , 10 , 11^In Brazil, long-term comparative data between CCTA and ICA in nonobstructive CAD remain limited.

The most commonly used imaging modalities for the diagnosis of CAD are invasive coronary angiography (ICA) and CCTA. Although these modalities differ substantially, few studies have directly compared them. The DISCHARGE trial^12^randomized 3,561 patients to ICA or CCTA and reported no significant differences in event rates between CCTA (2.1%) and ICA (3.0%) over a median follow-up of 3.5 years. Conversely, a meta-analysis by Xie et al.,^13^including more than 26,000 patients with stable CAD across six studies, suggested that ICA is associated with higher rates of major adverse cardiovascular events (MACEs) and all-cause mortality compared with CCTA.

In light of these conflicting findings, this study was designed to evaluate the comparative performance of ICA and CCTA in a large cohort of patients with nonobstructive CAD during long-term follow-up, with emphasis on clinical outcomes and factors associated with prognosis.

## Methods

### Population

Between 2011 and 2017, 4,004 consecutive patients aged 18-80 years, both men and women, from a specialized cardiology institution were evaluated. All participants had no prior coronary events and were referred for anginal symptoms (41%), positive functional tests for ischemia (17.5%), routine check-up (23%), or preoperative assessment (1.6%); in 16.9% of cases, the indication was unavailable. Patients were drawn from the hemodynamic Instituto do Coração registry and selected by convenience.

Exclusion criteria included prior acute coronary syndrome (ACS)/AMI, previous myocardial revascularization, valvular disease, cardiomyopathies, chronic obstructive pulmonary disease, chronic kidney disease, cancer, and hepatic failure.

The primary endpoint was a composite of all-cause mortality, ACS/AMI, and stroke. Secondary endpoints comprised the individual components of the primary endpoint. To reduce confounding in comparisons between ICA and CCTA, propensity score matching (PSM) was performed.^14^

The study was approved by the institutional Human Research Ethics Committee at Hospital das Clínicas, Universidade de São Paulo Medical School and registered at ClinicalTrials.gov (NCT05392491).

ICA and CCTA were performed by trained operators using standard techniques. Each case was independently reviewed by two trained technicians who analyzed reports generated by two experienced angiographers responsible for image interpretation. In cases of disagreement, records were reviewed by two senior cardiologists to reach consensus. Interobserver variability was 2.2% in 500 randomly selected cases using a computer-generated sequence (random.org). No procedure-related complications occurred.

Follow-up was conducted through clinic visits, telephone contact, or email. After informed consent, participants completed a structured questionnaire ( Appendix A ). Outcome adjudication was based on patient reports, family reports, medical records, or treating physician documentation.

Coronary arteries were classified as:

- No apparent CAD;
- Presence of CAD, classified according to a modified CAD-RADS™ 2.0 approach adapted for nonobstructive CAD. Lesion severity was defined as: no apparent (0%), mild (≤ 30%), and moderate (> 30%).

Plaque burden index was categorized as:

- No apparent: no lesion;
- Mild: up to two mild lesions (≤ 30%);
- Moderate: three mild lesions (≤ 30%) or up to two moderate lesions (> 30%);
- High: left main involvement or at least three moderate lesions (> 30%);
- Very high: greater lesion burden than previously classified as high, or at least one lesion ≥ 50% identified on a subsequent CCTA when available.

Coronary lesions were visually defined as the luminal diameter of diseased vessel compared to most normal appearing proximal segment. No complications occurred during CCTA.

### Demographics

Baseline data collected at study entry ( Table 1 ) included age, sex, weight, height, and blood pressure. Body mass index (BMI) was calculated as weight divided by height squared.

**Table 1:** – Baseline characteristics according to imaging modality (ICA vs CCTA)

Variable	ICA (n = 1,649)	CCTA (n = 2,355)	p-value	Total (%)
**Sex, n (%)**				
Women	956 (58.0)	1,080 (45.9)	< 0.001	2,036 (50.8)
Men	693 (42.0)	1,275 (54.1)	< 0.001	1,968 (49.2)
**Race, n (%)**				
White	1,148 (86.5)	1,790 (89.9)	> 0.05	3,208 (87.8)
**Age, n (%)**				
≤ 65 years	1,263 (76.6)	1,857 (78.9)	0.008	3,120 (77.9)
> 65 years	386 (23.4)	497 (21.1)	0.008	883 (22.1)
**Cardiovascular risk factors, n (%)**				
CAD family history	703 (52.9)	1,139 (57.0)	0.022	1,842 (55.4)
Diabetes mellitus	689 (41.8)	847 (36.0)	< 0.001	1,536 (38.4)
Dyslipidemia	1,082 (65.6)	1,690 (71.8)	< 0.001	2,772 (69.2)
Hypertension	1,355 (82.2)	1,673 (71.0)	< 0.001	3,028 (75.6)
Smoking	710 (44.7)	936 (41.1)	0.027	1,646 (42.5)
Alcohol consumption	358 (33.8)	910 (52.9)	0.001	1,268 (45.6)
Physical exercise	650 (41.7)	1,188 (53.1)	< 0.001	1,838 (48.4)
Obesity	434 (29.4)	594 (26.8)	0.001	1,028 (27.8)

CAD: coronary artery disease; CCTA: coronary computed tomography angiography; ICA: invasive coronary angiography.

Hypertension was defined as systolic blood pressure > 130/85 mmHg or use of antihypertensive medication. Obesity was defined as BMI ≥ 30, and overweight as BMI of 26-29.

Physical activity was categorized as sedentary or active (≥ three sessions per week). Positive family history was defined as at least one parent experiencing cardiovascular events or interventions before 60 years of age.

Smoking status was classified as never, former, or current (≥ 10 cigarettes/day). Diabetes mellitus was defined as fasting plasma glucose ≥ 126 mg/dL, 2-hour glucose tolerance test > 200 mg/dL, HbA1c ≥ 6.5%, or use of glucose-lowering medication. Dyslipidemia was defined as LDL > 130 mg/dL, triglycerides > 150 mg/dL, or use of lipid-lowering therapy.

Laboratory data are presented in Table 2 . No significant differences between groups were observed for any parameter except total cholesterol.

**Table 2:** – Laboratory data according to imaging modality (ICA vs CCTA)

Variable (mean ± SD)	ICA (n = 1,649)	CCTA (n = 2,355)	p-value	Total
**Urea (mg/dL)**	36.33 ± 11.46 (n = 732)	35.52 ± 10.24 (n = 1,187)	0.435	35.83 ± 10.72 (n = 1,919)
**Creatinine (mg/dL)**	0.95 ± 0.26 (n = 808)	0.94 ± 0.21 (n = 1,231)	0.922	0.94 ± 0.23 (n = 2,039)
**Sodium (mEq/L)**	140.36 ± 2.49 (n = 639)	140.30 ± 2.42 (n = 1,086)	0.687	140.32 ± 2.44 (n = 1,725)
**Potassium (mEq/L)**	4.32 ± 0.44 (n = 642)	4.32 ± 0.40 (n = 1,088)	0.612	4.32 ± 0.41 (n = 1,730)
**Total cholesterol (mg/dL)**	190.09 ± 42.30 (n = 740)	196.93 ± 49.63 (n = 1,114)	0.043	194.20 ± 46.95 (n = 1,854)
**HDL cholesterol (mg/dL)**	47.94 ± 14.19 (n = 738)	49.07 ± 14.29 (n = 1,113)	0.055	48.62 ± 14.26 (n = 1,851)
**Non-HDL cholesterol (mg/dL)**	142.30 ± 41.33 (n = 737)	147.84 ± 49.21 (n = 1,113)	0.153	145.63 ± 46.30 (n = 1,850)
**LDL cholesterol (mg/dL)**	115.39 ± 37.97 (n = 724)	119.88 ± 44.80 (n = 1,085)	0.273	118.08 ± 42.25 (n = 1,809)
**Triglycerides (mg/dL)**	133.64 ± 75.73 (n = 721)	143.75 ± 170.51 (n = 1,129)	0.489	139.81 ± 141.40 (n = 1,850)
**Glucose (mg/dL)**	113.77 ± 34.18 (n = 751)	113.11 ± 37.79 (n = 1,122)	0.045	113.37 ± 36.38 (n = 1,873)
**HbA1c (%)**	6.30 ± 1.28 (n = 481)	6.00 ± 1.04 (n = 919)	< 0.001	6.10 ± 1.14 (n = 1,400)

CCTA: coronary computed tomography angiography; HDL: high-density lipoprotein; ICA: invasive coronary angiography; LDL: low-density lipoprotein; SD: standard deviation.

### Statistical analysis

Categorical variables are presented as frequencies and compared using the chi-square test. Continuous variables with normal distribution are expressed as mean ± standard deviation and compared using unpaired *t* tests. Normality was assessed using Kolmogorov-Smirnov tests and histogram inspection.

Time from baseline examination to the primary endpoint was calculated for survival analyses. Event-free survival was estimated using Kaplan-Meier curves. Univariate and multivariable Cox regression analyses were performed when appropriate, and results are reported as hazard ratios (HRs) with 95% CIs.

To further control for confounders, PSM based on logistic regression was applied using a caliper of 0.01.^14^Matching included nine covariates: age, sex, blood pressure, dyslipidemia, smoking status, physical activity, diabetes, hypertension, and family history of CAD.

A significance level of 5% was adopted for all analyses. Statistical analyses were performed using IBM SPSS Statistics for Windows, version 25 (IBM Corp., Armonk, N.Y., USA).

## Results

### Baseline characteristics

Baseline demographic characteristics are summarized in Table 1 . A total of 4,004 patients were followed for a median of 9 years. The mean age was 57.9 ± 10.3 years. Overall, men and women were similarly represented; however, a higher proportion of men was observed in the CCTA group (54.1% vs 42.0%). CCTA was performed in 2,355 patients (58.8%) and ICA in 1,649 (41.2%).

Positive family history of CAD, dyslipidemia, and physical activity were more frequent in the CCTA group, whereas diabetes, hypertension, and smoking were more prevalent in the ICA group. Moderate or higher plaque burden at baseline was more common among ICA patients than among CCTA patients (68.2% vs 23.1%; p < 0.001, chi-square test) ( Appendix B ).

### Total number of events

During follow-up, 278 events (6.9%) occurred over 9 years, with a higher incidence in the ICA group compared with the CCTA group (9.4% vs 5.1%), predominantly among men older than 65 years. The absolute difference in event rates was 4.3%.

Death, stroke, and ACS/AMI were the most frequent events in the overall population and were significantly more common in the ICA group than in the CCTA group ( Table 3 ).

**Table 3:** – Number of clinical events according to imaging modality (ICA vs CCTA)

Endpoint	ICA n (%) (n = 1,649)	CCTA n (%) (n = 2,355)	Total n (%) (n = 4,004)	p-value
**Composite primary endpoint**	155 (9.4)	121 (5.1)	276 (6.9)	< 0.001
**Isolated endpoints**				
Death	72 (4.4)	44 (1.9)	116 (2.9)	< 0.001
Stroke	65 (3.9)	47 (2.0)	111 (2.8)	< 0.001
ACS/AMI	49 (3.0)	39 (1.7)	88 (2.2)	0.005

Individual components of the primary endpoint. ACS: acute coronary syndrome; AMI: acute myocardial infarction; CCTA: coronary computed tomography angiography; ICA: invasive coronary angiography.

### Survival and risk of events in patients with and without coronary artery disease

Overall, 37.3% of patients had no apparent CAD and 62.7% had nonobstructive CAD. Nonobstructive CAD was associated with significantly worse survival compared with no CAD ( Figure 1A ). Event-free survival was also worse among ICA patients than among CCTA patients ( Figure 1B ).

**Figure 1:** – Event-free survival according to CAD status and imaging modality (ICA vs CCTA). A) Event-free survival according to CAD status; B) event-free survival according to imaging modality; C) event-free survival among patients without apparent CAD; D) event-free survival among patients with nonobstructive CAD. CAD: coronary artery disease; CCTA: coronary computed tomography angiography; HR: hazard ratio; ICA: invasive coronary angiography.

Notably, ICA patients without apparent CAD showed significantly worse survival than comparable patients in the CCTA group ( Figure 1C ), suggesting that the two modalities provide different prognostic information despite similar classification as normal or no apparent CAD.

Furthermore, among patients with nonobstructive CAD identified by either ICA or CCTA, survival remained better in the CCTA group compared with the ICA group ( Figure 1D ).

### Plaque burden index vs clinical events

Figure 2 illustrates the relationship between plaque burden and clinical events. No statistically significant differences were observed between patients with low plaque burden and those without detectable lesions throughout follow-up.

**Figure 2:** – Event-free survival according to plaque burden index. HR: hazard ratio.

In contrast, patients with moderate and high plaque burden had a significantly higher risk of the primary endpoint compared with those without lesions, with similar risk between the moderate and high burden groups.

### Medications

Lipid-lowering therapy was more frequently used in the CCTA group, whereas antidiabetic agents, antihypertensive medications, and aspirin were more commonly used in the ICA group. Detailed results are provided in Appendix C .

### Determinants of clinical evolution

Unadjusted analyses identified 16 variables associated with outcomes. After adjustment, seven variables remained independently associated with events (p < 0.001): male sex, hypertension, age > 65 years, ICA group assignment, smoking, physical inactivity, positive family history of CAD, and plaque burden.

### Propensity score matching

Because this was not a randomized study, PSM was performed using age, sex, systolic blood pressure, dyslipidemia, smoking status, physical activity, diabetes, hypertension, and family history of CAD ( Table 4 ). A total of 1,186 matched pairs were identified.

**Table 4:** – Baseline characteristics after propensity score matching (ICA vs CCTA)

Characteristic	ICA (n = 1,187), n (%)	CCTA (n = 1,187), n (%)	p-value^**1**^
**Sex, n (%)**			0.019
Female	663 (55.9)	637 (53.7)	
Male	527 (44.1)	550 (46.3)	
**Age, n (%)**			0.955
≤ 65 years	920 (77.5)	918 (77.3)	
> 65 years	267 (22.5)	269 (22.7)	
**Cardiovascular risk factors, n (%)**			
CAD family history	631 (53.2)	636 (53.6)	0.828
Diabetes mellitus	465 (39.2)	499 (42.0)	0.080
Dyslipidemia	796 (67.1)	808 (68.1)	0.524
Hypertension	959 (80.8)	967 (81.5)	0.546
Smoking	511 (43.0)	484 (40.8)	0.155
Physical exercise	504 (42.5)	469 (39.5)	0.055

^1^McNemar test for paired samples. CAD: coronary artery disease; CCTA: coronary computed tomography angiography; ICA: invasive coronary angiography.

Among matched patients, male sex remained more prevalent in the ICA group, whereas positive family history was more frequent in the CCTA group. All other variables were balanced between groups.

After matching, survival remained lower in the ICA group compared with the CCTA group (HR 0.57; 95% CI 0.42-0.78; p < 0.001), suggesting that patients undergoing ICA had more advanced CAD.

## Discussion

In this long-term observational study, ICA and CCTA provided different prognostic information in nonobstructive CAD, which was associated with clinically relevant events. As expected, patients without CAD had better outcomes than those with nonobstructive CAD, regardless of the imaging modality. These findings corroborate previous studies showing that nonobstructive CAD is associated with substantial complications, including cardiovascular events, recurrent hospitalizations, and impaired quality of life.^16 , 17^

Notably, patients without apparent CAD in the ICA group had worse prognosis than comparable patients in the CCTA group. Moreover, across the overall cohort, individuals undergoing ICA demonstrated worse outcomes than those evaluated with CCTA. These results are consistent with prior evidence. Huang et al.^16^analyzed 64,905 patients undergoing ICA or CCTA, and Radico et al.^18^evaluated 34,039 patients with angina and nonobstructive CAD over 5 years; both studies concluded that nonobstructive CAD is associated with higher rates of MACEs compared with entirely normal coronary arteries. Indeed, Alfonso et al.^19^demonstrated using intravascular ultrasound that vessels considered normal by ICA may harbor atherosclerotic plaques. Additionally, Ludmer et al.^20^showed that patients with minimal coronary disease exhibit paradoxical vasoconstriction in response to acetylcholine, indicating endothelial dysfunction as a likely mechanism underlying events in patients with angiographically normal coronaries.^21 , 22^

Our findings are also consistent with the meta-analysis by Xie et al.,^13^which included 26,548 patients from six studies comparing ICA and CCTA. The authors reported that ICA was associated with higher risks of MACEs, all-cause mortality, and major procedure-related complications compared with CCTA, particularly during shorter follow-up (< 3 years). However, our results differ from those of the DISCHARGE trial,^12^which found no difference in event rates between ICA and CCTA over a median follow-up of 3.5 years. Although our study was not randomized, the substantially longer follow-up (9 vs 3.5 years) may explain this discrepancy. Importantly, propensity score analysis suggests that differences between modalities were not fully explained by measured confounders, except for positive family history, which was more common in the CCTA group, and male sex, which was more prevalent in the ICA group. Nevertheless, residual confounding cannot be excluded, particularly because imaging modality selection was determined by clinical judgment, likely favoring ICA in patients with more advanced disease considered for revascularization.

The discrepancy between ICA and CCTA observed in this study likely reflects intrinsic differences between the modalities. ICA primarily identifies luminal narrowing, whereas CCTA detects earlier atherosclerotic changes, including positive remodeling and coronary calcification, in addition to luminal stenosis. No intracoronary imaging or physiological assessment was performed. Although such information would be valuable, these techniques are not routinely used in daily clinical practice.

Plaque burden index was strongly associated with clinical events. Patients with no apparent CAD or mild plaque burden had similar outcomes, whereas moderate and high plaque burden were clearly associated with worse prognosis ( Figure 2 ). These findings align with prior studies demonstrating that event risk increases proportionally with the number of affected vessels, including extracoronary territories such as the carotid arteries.^23 - 25^In our cohort, higher plaque burden was associated with older age (> 65 years), consistent with previous observations in obstructive CAD.^5^In contrast to some prior reports, nonobstructive CAD was more frequent in men than women in our cohort, which may reflect population-specific characteristics.

Regarding pharmacological therapy, lipid-lowering medications were more frequently used in the CCTA group, whereas antidiabetic agents, antihypertensive drugs, and aspirin were more common in the ICA group. Medication use may have influenced outcomes; however, given the self-reported nature of these data and the long follow-up period, specific effects cannot be determined. Traditional risk factors, including dyslipidemia, hypertension, smoking, and physical inactivity, were present in more than half of the population, reinforcing their role as determinants of subclinical atherosclerosis. As with medication data, these variables should be interpreted cautiously due to reliance on long-term self-report.

### Strength and limitations

The main strengths of this study include the large sample size and the long observational follow-up (median 9 years), which, to the best of our knowledge, represents one of the longest follow-up periods reported in this context. The primary endpoint included only hard outcomes: all-cause mortality, stroke, and ACS/AMI. Furthermore, this pragmatic study reflects real-world clinical practice by evaluating ICA and CCTA, imaging modalities routinely used in daily cardiology care, thereby enhancing the clinical relevance of the findings.

Several limitations should be acknowledged. Data were self-reported, introducing potential bias, and the single-center design may limit generalizability. Although propensity score methods were used to mitigate confounding, the absence of randomization remains an important limitation. In particular, the choice of imaging modality by the treating physician may have influenced outcomes. Additionally, the lack of repeat imaging prevented assessment of atherosclerosis progression or plaque instability, which may have contributed to clinical events.

## Conclusion

Overall, our findings highlight important differences between ICA and CCTA in nonobstructive CAD during long-term follow-up and confirm that nonobstructive CAD is associated with significant clinical risk. Patients with nonobstructive CAD should not be reassured that they are normal or have clinically insignificant atherosclerosis. Imaging modality selection should be individualized according to clinical judgment. Functional ischemia testing and aggressive preventive strategies should be prioritized in this population. Early risk factor control, through pharmacological therapy and lifestyle modification, is essential.

## Supplemental Materials

Appendix A

Appendix B

Appendix C
